# Genetic Basis of Sexual Maturation Heterosis: Insights From Ovary lncRNA and mRNA Repertoire in Chicken

**DOI:** 10.3389/fendo.2022.951534

**Published:** 2022-07-27

**Authors:** Yuanmei Wang, Jingwei Yuan, Yanyan Sun, Yunlei Li, Panlin Wang, Lei Shi, Aixin Ni, Yunhe Zong, Jinmeng Zhao, Shixiong Bian, Hui Ma, Jilan Chen

**Affiliations:** Key Laboratory of Animal (Poultry) Genetics Breeding and Reproduction, Ministry of Agriculture, Institute of Animal Science, Chinese Academy of Agricultural Sciences, Beijing, China

**Keywords:** Heterosis, chicken, sexual maturation, ovary, lncRNA, WGCNA

## Abstract

Sexual maturation is fundamental to the reproduction and production performance, heterosis of which has been widely used in animal crossbreeding. However, the underlying mechanism have long remained elusive, despite its profound biological and agricultural significance. In the current study, the reciprocal crossing between White Leghorns and Beijing You chickens were performed to measure the sexual maturation heterosis, and the ovary lncRNAs and mRNAs of purebreds and crossbreeds were profiled to illustrate molecular mechanism of heterosis. Heterosis larger than 20% was found for pubic space and oviduct length, whereas age at first egg showed negative heterosis in both crossbreeds. We identified 1170 known lncRNAs and 1994 putative lncRNAs in chicken ovary using a stringent pipeline. Gene expression pattern showed that nonadditivity was predominant, and the proportion of nonadditive lncRNAs and genes was similar between two crossbreeds, ranging from 44.24% to 49.15%. A total of 200 lncRNAs and 682 genes were shared by two crossbreeds, respectively. GO and KEGG analysis showed that the common genes were significantly enriched in the cell cycle, animal organ development, gonad development, ECM-receptor interaction, calcium signaling pathway and GnRH signaling pathway. Weighted gene co-expression network analysis (WGCNA) identified that 7 out of 20 co-expressed lncRNA-mRNA modules significantly correlated with oviduct length and pubic space. Interestingly, genes harbored in seven modules were also enriched in the similar biological process and pathways, in which nonadditive lncRNAs, such as *MSTRG.17017.1* and *MSTRG.6475.20*, were strongly associated with nonadditive genes, such as *CACNA1C* and *TGFB1* to affect gonad development and GnRH signaling pathway, respectively. Moreover, the results of real-time quantitative PCR (RT-qPCR) correlated well with the transcriptome data. Integrated with positive heterosis of serum GnRH and melatonin content detected in crossbreeds, we speculated that nonadditive genes involved in the GnRH signaling pathway elevated the gonad development, leading to the sexual maturation heterosis. We characterized a systematic landscape of ovary lncRNAs and mRNAs related to sexual maturation heterosis in chicken. The quantitative exploration of hybrid transcriptome changes lays foundation for genetic improvement of sexual maturation traits and provides insights into endocrine control of sexual maturation.

## Introduction

Sexual maturation is a pivotal development stage and indispensable for achieving successful reproduction (1). The initiation of sexual maturation is complicated, which is contingent upon integrating different stimuli into neuroendocrine and endocrine signals along the hypothalamic-pituitary-gonadal (HPG) axis. HPG axis is shown to be under a dual control system with a stimulatory and an inhibitory branch (2), in which hypothalamic gonadotropin-releasing hormone (GnRH) played a central and positive role in activation of reproductive function (3, 4), whereas gonadotropin-inhibitory hormone (GnIH) have inhibitory effect on GnRH release and then delayed onset of sexual maturation. The release and secretion of GnIH are primarily under the influence of melatonin (MT). Heterosis refers to the superior performance of hybrid compared with their parent in production, fertility and adaptability (5, 6). It has been comprehensively exploited to improve yield and quality in plant and animal. The underlying mechanism has been studied for over century, but still remains elusive (7). As an importantly economic trait in chicken, sex maturation is controlled by HPG signals. Attaining sexual maturation at an early age was shown to increase total egg production (8, 9), which has been extensively utilized in crossbreeding in poultry industry. Therefore, it is of great value to unravel the molecular mechanism that regulates the heterosis of sexual maturation.

The superiority phenotype depends on the alteration of gene expression. In recent decades, genome-wide gene expression changes of hybrids relative to their parents have been illustrated in different species, such as microorganisms, plants and animals. The transcriptional changes in hybrids generally associated with processes involved in the energy production, metabolic rates, stress response and hormone signaling (10). In chickens, multiple studies illustrated the heterosis mechanism of important traits at different stages through genome-wide expression analysis of brain, liver and muscle (11–13). Long non-coding RNAs (lncRNAs) are pivotal epigenomic factors, a class of endogenous more than 200 bases in length, and could affect gene expression by acting as cis- or trans- regulators (14). It was reported that lncRNA could involve in sexual maturation through regulating oogenesis and folliculogenesis (15). Recent study in C. elegans found the lncRNAs *let 7* could schedule sexual maturation through regulating their target genes (16). Moreover, Shu et al. (17) profiled lncRNA expression of pepper leaves and concluded that lncRNAs participated in seedling and flowering heterosis. Taken together, the integrated analysis of lncRNA and mRNA may provide novel clues for exploring the mechanism of sexual maturation heterosis.

Chicken meat and egg are great protein sources for human. Heterosis utilization is routine for economic traits improvement in poultry breeding (18). It was documented that negative heterosis for age at first egg (AFE) was observed in hybrids of different chicken breeds (19, 20). Moreover, the expression of ER and FSHR was reported to be correlated with AFE heterosis in chicken (21). Nevertheless, previous study mostly concentrated on exploring heterosis mechanism through characterizing several candidate genes. The genome-wide gene expression of sexual maturation heterosis was rarely reported and investigated. In this study, White Leghorn and Beijing You chicken were employed as parents to generate purebreds and reciprocal crossbreeds, respectively. The White Leghorn is a dominant commercial layer breed around the world. The Beijing You chicken is a traditional Chinese indigenous breed with head crest, beard and fuzzy shank, which produced much less egg mass compared to the White Leghorn. These two breeds have previously been reported genetically distant (22), and this would contribute the heterosis of traits. The transcriptome analysis of the ovary in purebreds and crossbreeds was implemented to reveal the key lncRNAs and genes regulating the formation of sexual maturation heterosis. Our study would provide new insights into the molecular basis of sexual maturation heterosis in chicken, and lay theoretical foundation for effectively utilization of heterosis.

## Materials and Methods

### Ethics Consideration

All experimental procedures involving the use of animals were conducted according to the Guidelines for Experiment established by the Science Research Department of the Institute of Animal Sciences, Chinese Academy of Agriculture Sciences (IAS-CAAS), and ethical approval was received from the Animal Ethics Committee of the IAS-CAAS (no. IAS 2022-35).

### Experimental Populations

In the present study, White Leghorn and Beijing You chicken were employed as parents to generate purebreds (WW and YY) and reciprocal crossbreeds (WY and YW). Briefly, 30 males and 150 unrelated females from White Leghorn, and 30 males and 150 unrelated females from Beijing You chicken were selected for mating to generate WW and YY and for reciprocal mating to generate WY and YW based on laying consistency, semen quality and body weight. Chickens from a single hatch were raised under the same environment with free access to feed and water in the experimental farm of IAS-CAAS.

### Phenotype Measurement and Sample Collection

Egg was collected for each individual daily from the age at first egg (AFE). When the egg laying ratio of population reached 5%, thirty chickens from each group were randomly selected to measure pubic space. Meanwhile, six individuals from WW, YY, WY and YW were selected based on the average of pubic space of each group, respectively. Subsequently, all visible follicles were carefully excluded when ovary tissue was collected as previously reported (23). Then, the ovary was frozen immediately in liquid nitrogen for RNA sequencing. In addition, the oviduct was captured for length measurement by image J (24). Heterosis of phenotype was calculated according to the following equation:


$$\text{H}\%=\frac{\bar{{\text{F}}_{1}}-(\bar{{\text{P}}_{\text{M}}}+\bar{{\text{P}}_{\text{F}}})/2}{(\bar{{\text{P}}_{\text{M}}}+\bar{{\text{P}}_{\text{F}}})/2}\times 100\%$$

Where $\bar{{\text{F}}_{1}}$ , $\bar{{\text{P}}_{\text{M}}}$ and $\bar{{\text{P}}_{\text{F}}}$ are the mean phenotype of reciprocal crossbreeds, maternal and purebreds, respectively. In addition, Student’s *t*-value was estimated to evaluate the significance of H% based on the formula of Wu and Zhang (25):


$$\text{t}=\frac{\text{H}\%}{2\sqrt{\frac{\sum {\left({\text{F}}_{1\text{i}}-\bar{{\text{F}}_{1}}\right)}^{2}}{\text{N}-1}}/\left[\left(\bar{{\text{P}}_{\text{M}}}+\bar{{\text{P}}_{\text{F}}}\right)\times \sqrt{\text{N}}\right]}$$

where F_1i_ is the phenotype of individual i in crossbreeds; N is the number of chickens in WY or YW. *P* value was obtained according to the student’s *t*-test and degrees of freedom. H% was considered statistically significant if *P* < 0.05.

### RNA Extraction, Library Construction and Sequencing

Total RNA was extracted using TRIzol^®^ Reagent (Invitrogen, USA) following the manufacturer’s instructions. The RNA integrity and quality were determined by agarose gel electrophoresis and NanoPhotometer^®^ spectrophotometer (IMPL EN, CA, USA). Three micrograms of total RNA were used for the construction of lncRNA library, and then the ribosomal RNA was removed from total RNA with TruSeq Stranded Total RNA Library Prep Gold Kits (Illumina, United States). Finally, 24 RNA sequencing libraries were constructed for paired-end sequencing according to the instructions of NEB Next^®^ Ultra Directional RNA Library Prep Kit for Illumina^®^ (NEB, Ipswich, MA, USA). RNA-Seq were performed using Novaseq6000 (Illumina, San Diego, United States) to generate 150bp paired-end reads.

### Data Quality Control and Assembly

The reads, including adapter contamination, low-quality reads (Phred Quality Score < 5%), reads with poly-N > 5% and reads matched rRNA were filtered out using in-house perl scripts to generate clean reads, which were aligned to the chicken reference genome (http://ftp.ensembl.org/pub/release-106/fasta/gallus_gallus/) using HiSAT2 (v2.1.0) (26) with default parameters, and then the mapped reads was assembled by StringTie (v2.1.5) (26) with gene transfer format (GTF) file of Ensembl gene annotation (http://ftp.ensembl.org/pub/release-106/gtf/gallus_gallus/). According to the chicken gene annotation, the number and proportion of three lncRNA functional elements (exon, intron and intergenic) were calculated based on the unique mapping sequences.

### LncRNA Identification

The potential lncRNAs were winnowed with the following criteria. Firstly, the transcripts less than 200 bp and without strand information were removed. Secondly, transcripts with class code “i”, “u” and “x” were retained. Subsequently, the sequence of known lncRNAs were download from two lncRNA databases, Domestic-Animal LncRNA Database (ALDB) (12) and NONCODE (27). We then used BLAST to align the unannotated transcripts to known lncRNAs. The transcripts perfectly aligned with sequences in either ALDB or NONCODE were regarded as known lncRNAs. Then, the protein coding potential of the remaining transcripts was predicted using CNCI (28), CPC (29), PLEK (30) and Pfam (31) software. Only transcripts with CNCI score < 0, CPC score < 0, PLEK score < 0 and Pfam E-value < 1e-5 were established as putative lncRNAs. Finally, the cis- and trans- acting relationship between lncRNAs and potential protein-coding genes was predicted according to their distances and expression correlations (32).

### Differential Inheritance Patterns of lncRNAs and Genes

Differentially expressed lncRNAs (DELs) and differentially expressed genes (DEGs) between two different groups were analyzed based on gene counts by DESeq2 (v1.16.1) (33) in R project. Meanwhile, |Log2(fold change) | >1 and adjusted *P* value < 0.05 were taken as criteria to identify the DELs and DEGs in the corresponding comparison. These genes were further classified into three inheritance patterns: additivity, dominance and overdominance, based on the gene expression level of purebreds and crossbreeds as previously reported (34). Briefly, additivity (IV and X) occurred when gene expression was significantly different between two purebreds, and gene expression of crossbreeds was higher than one purebred but lower than the another. Gene expression within crossbreeds that was not significantly different from one purebred but significantly higher (or lower) than the another was regarded as dominance (II, IV, IX, and XI). Gene expression within crossbreeds that was significantly higher (or lower) than both purebreds was considered as overdominance (V, VI, VIII, III, VII, and X).

### LncRNA and mRNA Co-Expression Network Analysis

The weighted gene co-expression network analysis (WGCNA) was performed to identify co-expressed modules of highly correlated genes and summarized such modules based on the correlation between module eigengene and phenotypes (35). Briefly, an expression matrix of all lncRNAs and mRNAs was constructed. The top 40% most variant genes were used for subsequent analysis. After the weighted adjacency matrix established, an unsigned weighted correlation network was constructed by creating a matrix of Pearson correlation coefficients of each pair genes. Then, each topological matrix was used as input for linkage hierarchical clustering analysis, and primary gene modules was detected using a dynamic tree cutting algorithm (deepSplit = 2, minModuleSize = 50, mergeCutHeight = 0.25). Subsequently, module eigengenes were correlated with different traits and searched against the most significant correlations. Finally, the lncRNAs-mRNAs networks were visualized with Cytoscape (v.3.8.0), which was run with layout “attribute circle layout”.

### Functional Annotation and Pathway Enrichment Analysis

To investigate biological function of nonadditive genes, we performed functional enrichment analysis, including Gene Ontology (GO) and Kyoto Encyclopedia of Genes and Genomes (KEGG) pathways *via* KOBAS 3.0 platform (36). The GO terms and KEGG pathways with *P* value < 0.05 were considered significantly enriched.

### GnRH and MT Content Assay

GnRH and MT content was detected using the GnRH and MT assay kit (Beijing Laibotairui Technology Co. Ltd, Beijing, China) following the manufacture guidelines, respectively. Briefly, serum (40 μL) from each chicken was added to each well of 96-well plate. Then, the 96-well plate was incubated for one hour at 37°C. Following washed five times with washing liquid, color liquid A and B was added successively. After incubating for 10 min at 37°C, 50 μL of termination solution was added. Finally, the OD value was detected at 450 nm by Thermo Multiskan Ascent.

### RT-qPCR Validation

One microgram total RNA of each sample was reverse transcribed into cDNA using the PrimeScript RT Reagent Kit (TaKaRa, Shiga, Japan). The specific primer sequences were designed using Primer Premier5.0. The RT-qPCR reaction mixture (10 µL) consisted of cDNA 1.5 µL, forward primer 0.5 µL (10µM), reverse primer 0.5 µL (10µM), SYBR Green Master (Mix) 5 µL and ddH_2_O 2.3 µL. The amplification condition was as follows: 95°C for 3 min, followed by 40 cycles at 95°C for 3 s, 60 °C for 32s, finally, a single melt cycle was 95°C for 15 s and 65°C for 1 min. Triplicate was performed for each sample using PrimeScript One Step RT-PCR Kit (TaKaRa, Shiga, Japan). The results were normalized to GAPDH expression.

### Statistical Analysis

Fold changes in gene expression were calculated using the 2^−ΔΔCt^ method. All data are presented as the mean ± standard deviation (SD). The correlation between results of RT-qPCR and RNA-seq was calculated using Pearson^’^s correlation method in R (v.4.0.2).

## Results

### Heterosis of Pubic Space, Oviduct Length and Age at First Egg

**Figure 1:**
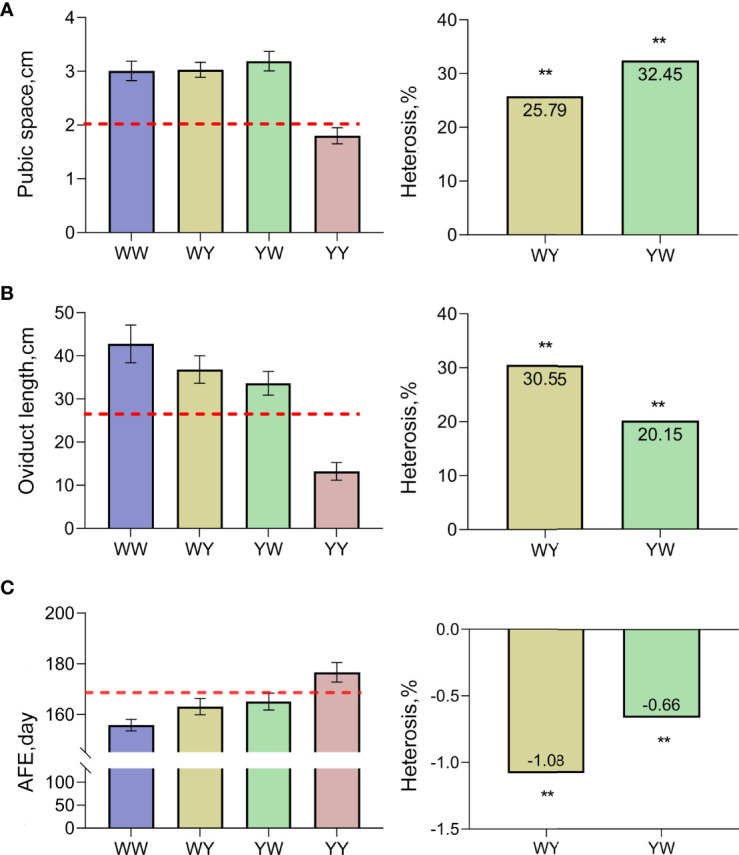
Heterosis of traits related to sexual maturation. **(A)** Heterosis of pubic space. **(B)** Heterosis of oviduct length. **(C)** Heterosis of AFE. The dashed line represents mid parent value. ** indicate that heterosis was highly significant (*P* < 0.01), respectively.

As shown in **Figure 1A**, the pubic space of crossbreeds was larger than the average value of purebreds, and significant positive heterosis (25.79%, 32.45%) was observed in both WY and YW (*P* < 0.05). Similarly, the larger oviduct length was observed in WY and YW compared with the average length of purebreds (**Figure 1B**), and heterosis of oviduct length was 30.55% in WY and 20.15% in YW, respectively. Moreover, AFE in crossbreeds was earlier than the average age of purebreds exhibiting negative heterosis (**Figure 1C**).

### Identification and Characterization of lncRNAs

**Figure 2:**
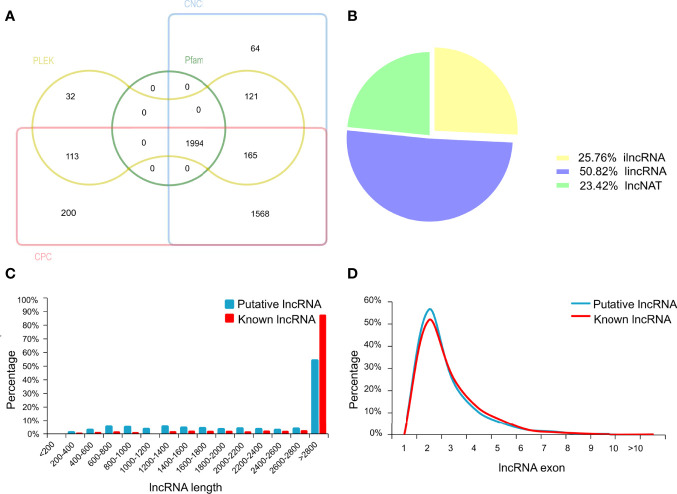
The identification of long non-coding RNAs (lncRNAs) in chicken ovary. **(A)** Venn diagram analysis showing the number of common and unique novel lncRNAs identified by CNCI, CPC, PLEK and Pfam database. **(B)** Classification of the uniquely mapped read locations including exon, intron and intergenic regions. ilncRNA, intergenic lncRNA; lincRNA, intron lncRNA; lncNAT, antisense lncRNA. **(C)** Length of known and novel lncRNAs. **(D)** The percentage of exon number for known and novel lncRNAs.

In the present study, an average of 36,579,837 raw reads was generated from each library, with average Q30 bases higher than 94.56% (**Supplementary Table S1**). The percentage of uniquely mapped reads and multi-mapped reads was 82.13% and 2.73%, respectively. In total, we characterized 3164 lncRNAs in chicken ovary (details in **Supplementary Table S1**). Among these lncRNAs, 1170 known lncRNA were identified through aligning lncRNAs sequence to the NONCODE or ALDB database. Besides known lncRNAs, a total of 1994 transcripts were identified as putative lncRNAs by CNCI, CPC, PLEK and Pfam software (**Figure 2A**). The majority of putative lncRNA were intergenic (50.82%), and 25.76% were intron, and 23.42% were antisense lncRNAs (**Figure 2B**). Furthermore, the length of the putative lncRNAs was mainly ranging from 2,800 to 3,000bp, similar to that of the known lncRNAs in ovary tissue (**Figure 2C**). The distributions of putative and known lncRNAs on the chromosomes were varied (**Supplementary Figure S1**). Moreover, the number of lncRNAs located on chromosome 1 was the largest, which accounted for 8.93% in putative lncRNAs and 13.08% in known lncRNAs. The most putative and known lncRNAs possessed two exons. The exon numbers of putative lncRNAs were greater than known lncRNAs (**Figure 2D**).

### Inheritance of lncRNAs and mRNAs

**Figure 3:**
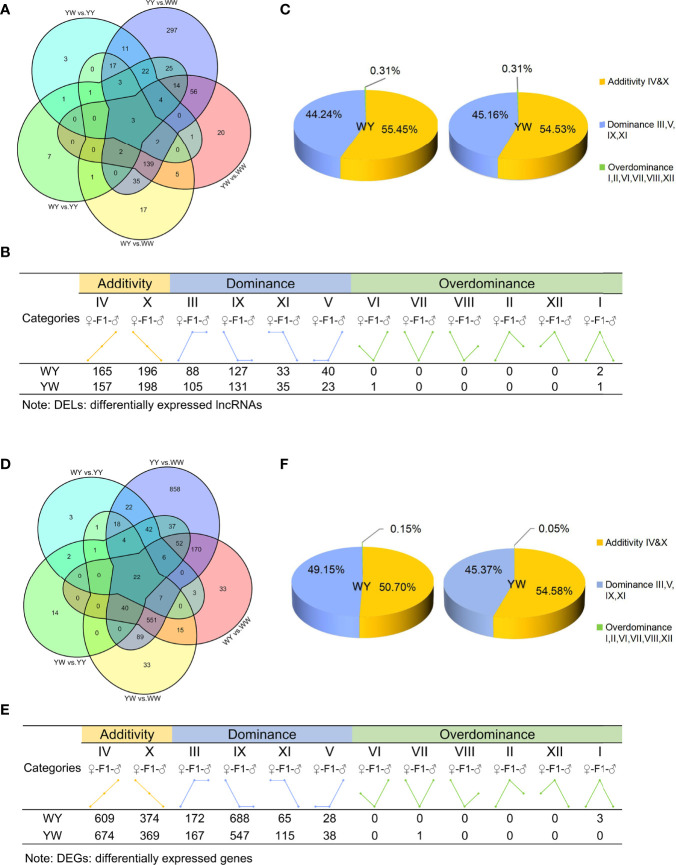
Analysis of lncRNAs and genes inheritance patterns. **(A)** The number of differentially expressed LncRNAs (DELs) among purebreds and crossbreeds. **(B)** Inheritance patterns of DELs between crossbreeds. DELs were divided into 12 types, e.g., class I, II, III, IV, V, VI, VII, VIII, IX, X, XI, XII, and further classified into three inheritance patterns: additivity (class IV and X), dominance (class III, V, IX and XI) and overdominance (class I, II, VI, VII, VIII, XII), based on the level of gene expression exhibited by purebreds and crossbreeds. Additivity, dominance and overdominance are presented in orange, blue and green, respectively. **(C)** The proportion of DELs in additivity, dominance and overdominance pattern. **(D)** The number of DEGs among purebreds and crossbreeds. **(E)** Inheritance patterns of DEGs of crossbreeds. DEGs were divided into 12 types, e.g,. class I, II, III, IV, V, VI, VII, VIII, IX, X, XI, XII, and further classified into three inheritance patterns: additivity (class IV and X), dominance (class III, V, IX and XI) and overdominance (class I, II, VI, VII, VIII, XII), based on the level of gene expression exhibited by crossbreeds and purebreds. Additivity, dominance and overdominance are presented in orange, blue and green, respectively. **(F)** The proportion of DEGs in additivity, dominance and overdominance pattern.

A principal component analysis (PCA) was performed to visualize the group differences of gene expression. The purebreds and crossbreeds were obviously separated from each other (**Supplementary Figure S2**), indicating significant differences of gene expression between two purebreds and between purebreds and crossbreeds. With the criteria of |log2 (fold change) | > 1 and adjusted *P* value < 0.05, we identified 686 DELs between two purebreds and between purebreds and crossbreeds (**Figure 3A**), including 630 in YY vs. WW, 225 in WY vs. WW, 83 in WY vs. YY, 246 in YW vs. WW and 68 YW vs. YY (**Supplementary Table S2**). These DELs were clustered into 12 types (I, II, III, IV, V, VI, VII, VIII, IX, X, XI and XII, details in **Supplementary Table S3**) according to their expression in purebreds and crossbreeds. The 12 types were further clustered into three main inheritance patterns: additivity (IV, X), dominance (III, V, IX, XI) and overdominance (I, II, VI, VII, VIII, XII). The number of dominant lncRNAs was 288 in WY and 294 in YW, respectively. The number of overdominant lncRNAs was 2 and 2 in WY and YW, respectively. The nonadditive lncRNAs accounted for 44.24% in WY and 45.16% in YW, respectively (**Figures 3B, C**). Additionally, a total of 200 nonadditive lncRNAs were shared by WY and YW (**Supplementary Figure S3A**). The number of unique nonadditive lncRNAs was 90 in WY and 96 in YW, respectively.

**Figure 4:**
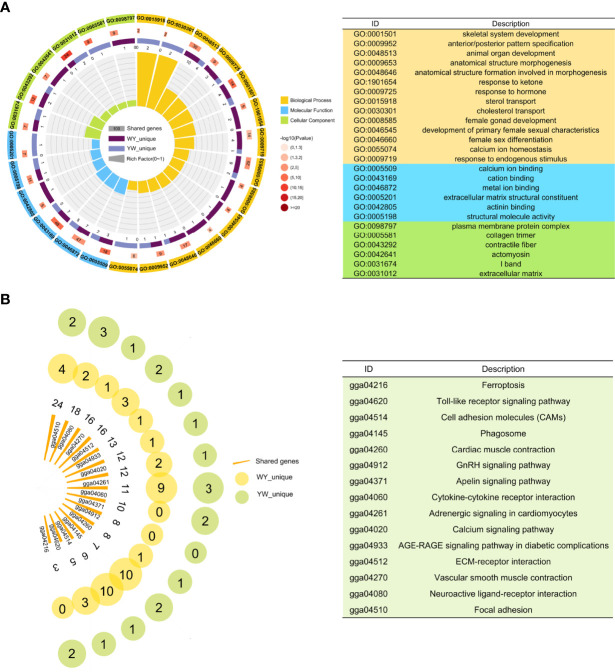
Function enrichment analysis of nonadditive genes. **(A)** Significant GO terms of nonadditive genes in crossbreeds. From outer to inner, the outermost circle represents the IDs of enriched GO terms. The names of GO ID in orange, blue and green represent biological process, molecular function and cell composition respectively. The second circle indicates shared genes enriched in GO terms. In the third circle, the piece in dark purple and light purple represents unique genes of WY and YW, respectively. In the innermost circle, each bar represents one GO term, and the size represents the rich factor. **(B)** KEGG pathway analysis for nonadditive genes. Each bar represents one pathway and the size represents the number of shared genes. The yellow and green circle represents the unique genes in WY and YW of each pathway, respectively.

There were 2023 DEGs between two purebreds and between crossbreeds and purebreds identified (**Figure 3D**), including 1918 in YY vs. WW, 899 in WY vs. WW, 131 in WY vs. YY, 781 in YW vs. WW and 220 YW vs. YY (**Supplementary Table S2**). These DEGs were clustered into 12 types, and the gene number of different patterns in reciprocal crossbreeds was shown in **Figure 3E**. The dominant genes were 956 in WY and 868 in YW, respectively. The number of overdominant genes was 3 and 1 in WY and YW, respectively. The nonadditive genes, accounted for 49.30% in WY and 45.42% in YW (**Figure 3F**). Among the nonadditive genes, a total of 682 genes were shared by WY and YW, and the number of unique genes was 274 in WY and 186 in YW (**Supplementary Figure S3B**), respectively. As shown in **Figure 4A**, gene ontology (GO) analysis showed that the nonadditive genes were mainly enriched in three categories, including growth and development related GO terms, such as animal organ development, gland morphogenesis, female gonad development and female sex differentiation, ion transport related GO terms, such as calcium ion transport, calcium ion homeostasis and metal ion transport, and other GO terms, such as response to hormone, steroid binding, response to endogenous stimulus and sterol transport (details in **Supplementary Table S4**). Interestingly, the majority of GO terms were also enriched by shared nonadditive genes. Given that the nonadditive genes of WY and YW were also significantly enriched in the biological process of female gonad development, we further characterized the common nonadditive genes harbored in the GO terms shared by WY and YW. The four common nonadditive genes, including transforming growth factor beta 1 (*TGFB1*), collagen type VI alpha 1 chain (*COL6A1*), CCAAT/enhancer binding protein beta (*CEBPB*) and receptor tyrosine kinase (*KIT*) genes, were involved in the growth and development of ovarian cells (**Supplementary Table S4**).

KEGG pathway analysis showed that nonadditive genes were significantly enriched in the focal adhesion, ECM-receptor interaction, vascular smooth muscle contraction, GnRH signaling pathway, calcium signaling pathway and folate biosynthesis (**Figure 4B**). These pathways were also enriched by shared nonadditive genes. Regarding the GnRH signaling pathway, we found eight common nonadditive genes including *G protein subunit alpha q* (*GNAQ*), *calcium voltage-gated channel subunit alpha1 C* (*CACNA1C*), *ENSGALG00000005884*, *adenylate cyclase 6* (*ADCY6*), *calcium voltage-gated channel subunit alpha1 D* (*CACNA1D*), *ENSGALG00000008727*, *early growth response 1* (*EGR1*) and *matrix metallopeptidase 2* (*MMP2*) were enriched in the pathway (**Supplementary Table S4**).

### Analysis of Co-Expressed Gene Networks and Candidate Genes

**Figure 5:**
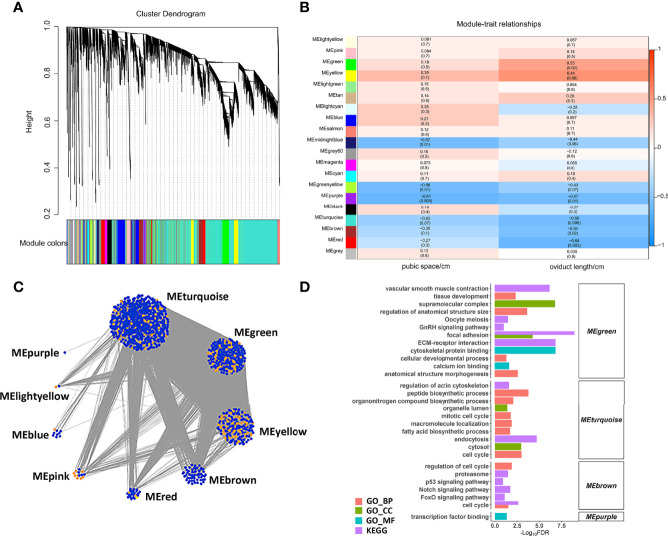
WGCNA analysis of lncRNAs and mRNAs. **(A)** Hierarchical cluster of 20 modules co-expressed mRNAs and lncRNAs. **(B)** Module-trait relationships. Each row represents a module eigengene, and each column present a trait. Each module includes the corresponding correlation and *P* value. **(C)** The networks of top 1% lncRNAs and mRNAs in nine selected modules. The orange and blue dots represent the hub lncRNAs and mRNAs, respectively. **(D)** The enriched function of each module.

We constructed a co-expression network of lncRNAs and mRNAs to infer the underlying regulatory function and potential co-expressed genes and lncRNAs. Correlated lncRNAs and mRNAs were clustered into 20 modules by WGCNA, and each module contained at least 50 genes (**Figure 5A**). Based on the correlation between the clusters and phenotypes, seven modules were significantly correlated with pubic space or oviduct length (*P* < 0.05) (**Figure 5B**). Among these modules (ME), the midnightblue ME (r = -0.57, p = 0.01) and greenyellow ME (r = -0.56, p = 0.01) were negatively correlated with pubic space. The purple ME was negatively correlated with pubic space (r = -0.61, p = 0.005) and oviduct length (r = -0.57, p = 0.01). The green ME was positively correlated with the oviduct length (r = 0.53, p = 0.02). The turquoise ME (r = -0.59, p = 0.008), the brown ME (r = -0.54, p = 0.02) and the red ME (r = -0.64, p = 0.003) was negatively correlated with oviduct length. Additionally, for all lncRNAs and protein coding genes harbored in the co-expression network, the top 1% of highly correlated gene pairs were chosen to visualize the correlation of modules (**Figure 5C**). Function enrichment results of each cluster suggested that the highly correlated lncRNAs and genes were mainly enriched in the developmental related process. Notably, the annotated genes in cluster green ME, were significantly enriched in oocyte meiosis, ECM-receptor interaction, calcium ion binding and GnRH signaling pathway, suggesting that lncRNAs in this cluster is essential for those biological processes. In addition, genes in turquoise ME were found closely correlated with fatty acid biosynthetic process, cell cycle and peptide biosynthetic process (**Figure 5D**).

**Figure 6:**
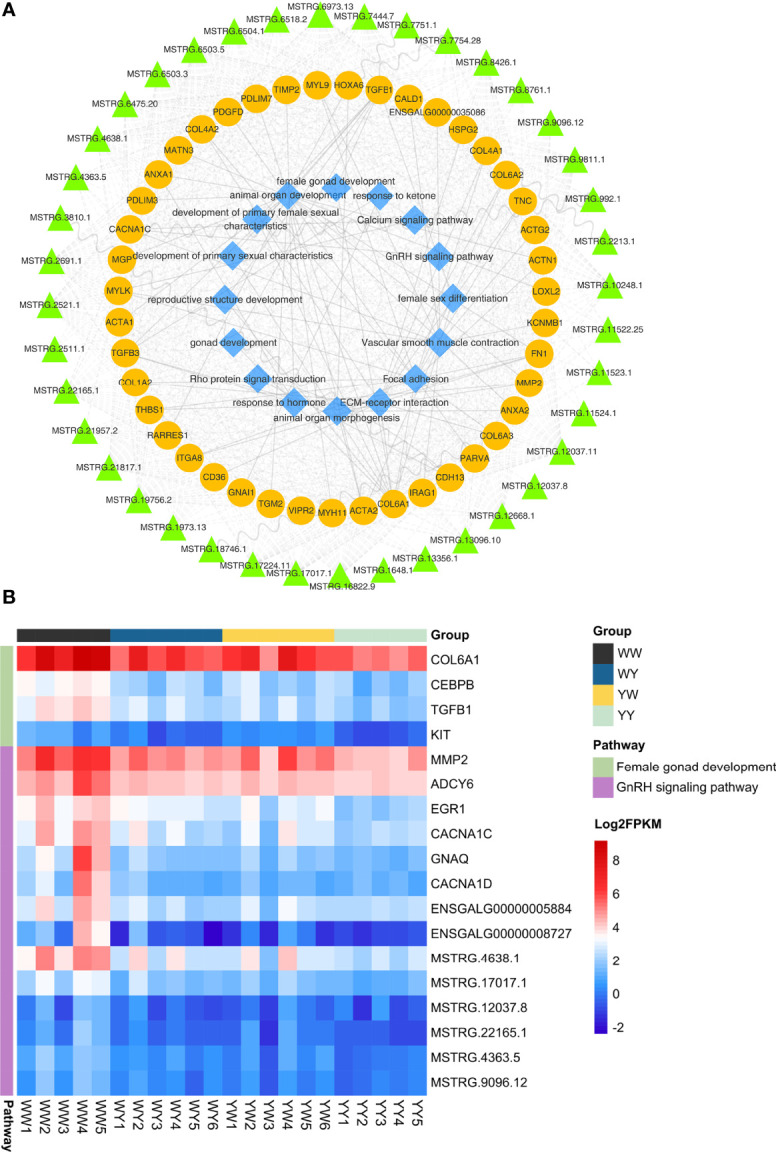
Co-expression network analysis of the paired lncRNA-mRNA involved in sexual maturation. **(A)** Network of lncRNAs, mRNAs and function. The green triangle, yellow circles and blue diamond represent lncRNAs, mRNAs and function, respectively. **(B)** The expression of nonadditive mRNAs and their lncRNAs enriched in GnRH signaling pathway and female gonad development.

We found 43 nonadditive genes were interactive and harbored in the seven associated modules, and these genes were regulated by 40 nonadditive lncRNAs in cis or trans (**Figure 6A**). Interestingly, function of these nonadditive genes were associated with reproductive structure development, gonad development, female gonad development, calcium signaling pathway and GnRH signaling pathway. The number of common nonadditive genes enriched in female gonad development and GnRH signaling pathway was four and eight in crossbreeds (**Figure 6B**), respectively. Moreover, *CNCAN1C* and *TGFB1*, which were regulated by *MSTRG.17017.1* and *MSTRG.6475.20* in trans, were enriched in GnRH signaling pathway and female gonad development, respectively. Accordingly, we found the expression of FSHR and ESR2 in crossbreeds was both higher than that of purebreds. But these two genes were not nonadditive genes (**Supplementary Figure S4**).

### GnRH and MT content

**Figure 7:**
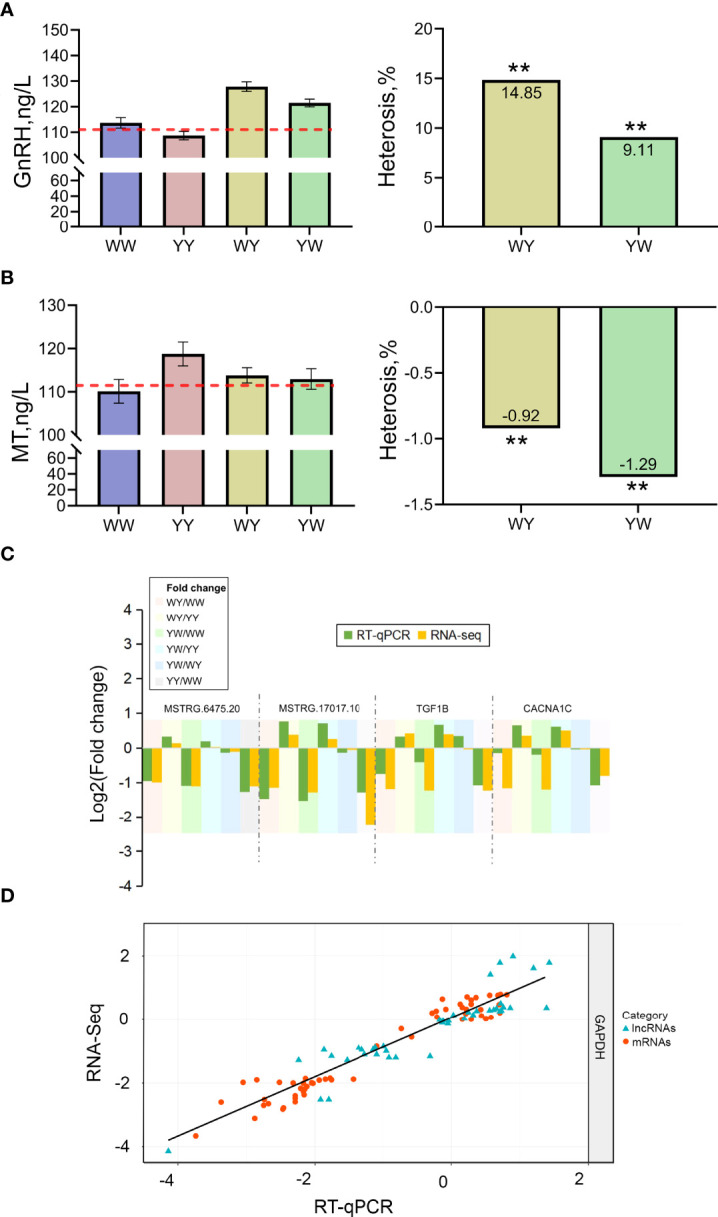
Validation of hormone concentration and gene expression. **(A)** The content of GnRH. **(B)** The content of MT. ** indicated adjusted *P* value less than 0.01. **(C)** The expression of candidate lncRNAs and mRNAs. **(D)** Correlation of gene expression level of 13 differentially expressed genes (DEGs) and six differentially expressed lncRNAs (DELs) using RNA-Seq and RT- qPCR. The x- and y- axis represents the log2 (fpkm) measured by RNA-Seq and RT-qPCR, respectively. GAPDH was used as reference gene. The blue and red dots represent the DEGs and DELs, respectively.

The nonadditive genes were related to GnRH signaling pathway and female gonad development, implying that hormone secretion plays a vital role in positive heterosis of sexual maturation. Then, the serum GnRH and MT concentration were detected. GnRH content in crossbreeds was significantly higher than the average value of purebreds, and showed positive heterosis (*P* < 0.05) (**Figure 7A**). MT content was lower in reciprocal crossbreeds compared with the average value of purebreds exhibiting negative heterosis (**Figure 7B**).

### RT-qPCR

To validate the RNA sequencing results, the expression of 13 DEGs and 6 DELs was detected using RT-qPCR. The specific primers for genes and lncRNAs were listed in **Table 1**. The expression of DEGs and DELs between any two groups showed consistent trend with the results of RNA-seq. A high correlation coefficient of gene expression level was detected (R^2^ = 0.91), indicating that RNA-seq data were reliable (**Figures 7C, D**).

**Table 1:** Primers for differentially expressed mRNAs and lncRNAs used in RT-qPCR.

Gene ID	Gene Symbol	Direction>	Sequence
ENSGALG00000014442	GAPDH	Forward	ATCACAGCCACACAGAAGACG
		Reverse	TGACTTTCCCCACAGCCTTA
ENSGALG00000000402	LOXL2	Forward	ACGGAGGGTTACGTGGA
		Reverse	AATGCTGCTTCCTTCTGCT
ENSGALG00000012200	GCH1	Forward	CCAGGAACGCCTTACCA
		Reverse	ATTTTCTGTACCCCACGCA
ENSGALG00000001573	P2RX1	Forward	AGCATCACCTTCCCCAA
		Reverse	GCTCAAACATAGGGCACAA
ENSGALG00000006190	FHL1	Forward	ATGCTGAGGGAGAACAACA
		Reverse	CAGTCCTTATGCCAGACCA
ENSGALG00000042458	ACTN1	Forward	GCTTCTACCACGCCTTCTC
		Reverse	TCCACTCCAGCAGATCACT
ENSGALG00000000318	CSRP1	Forward	AACCCGAACGCATCCAGAAT
		Reverse	ACTTGTGCCAGGACTTTCCA
ENSGALG00000042836	KCNH2	Forward	CTACTTCATCTCGCGTGGCT
		Reverse	AATGTCGTTCTTCCCCAGGA
ENSGALG00000003521	TPM1	Forward	GAAGAGAAAGTGGCGCAT
		Reverse	CGGCAAGCAGGTGTAAA
ENSGALG00000039742	CYP21A1	Forward	ATGAGTTCCTGCCCGAGCG
		Reverse	AATCGCTGTAGGATGTGCCC
ENSGALG00000040655	FAM20A	Forward	GACTGCAGCCAGATTGTGAAG
		Reverse	TTGTGCCGCTGGAAGTCTAC
ENSGALG00000013022	CACNA1C	Forward	GGAAGCAAGCGGAACTC
		Reverse	GGCAGGGTAACAAACCAG
ENSGALG00000031325	TGFB1	Forward	ACCCGATGAGTATTGGGCCAAAGA
		Reverse	GCGGGACACGTTGAACACGAA
MSTRG.4638.10		Forward	GCGGGGAAAACGCTCTTACTT
		Reverse	GATGCCTGACGGTGTGGAGG
MSTRG.6475.20		Forward	AAATGTCCTCACCCAGGCAG
		Reverse	CGGCAATTCACAGTTTGGTTCT
MSTRG.8761.10		Forward	CGAGGTTTTTCTGCGCTTGA
		Reverse	GATTTCCCCTCTCGGTTCCC
MSTRG.9096.12		Forward	CAATGTGCTTATGTTTCTCAGCA
		Reverse	AGCTGCCGTACACAAATCAA
MSTRG.17017.10		Forward	GTGCCAGCCAAAACAGGACA
		Reverse	TCCCAGAGCCTAACTCTTCCA
MSTRG.12075.10		Forward	CGCCCATGAAACCCTGATTG
		Reverse	CCATTCCCCATCTCTACGCC

## Discussion

The utilization of sexual maturation heterosis has contributed to egg production improvement in chicken for decades. However, our understanding of its molecular basis is still rudimentary, constraining its flexible and accurate application. In the current study, we found negative heterosis of AFE in both crossbreeds. Similar results were also reported in hybrids of other chicken breeds (37, 38). Female sexual maturation is a complicated biological process accompanying with the growth and development of gonad and internal reproductive organs. Pubic space and oviduct length are important indicators of gonad development in chickens (39). Significantly positive heterosis for both traits were observed in crossbreeds, implying the superiority of sexual maturation in hybrids. Accumulating transcriptome analysis focused on heterosis of different traits were conducted, which facilitated the understanding of the molecular mechanism of hybrid vigor in different tissues of various species (10). Hence, the comprehensive gene expression patterns in purebreds and crossbreeds were profiled to reveal the potential mechanisms of sexual maturation heterosis. To our knowledge, the genome-wide expression of lncRNAs and mRNAs related to sexual maturation heterosis in chicken was firstly characterized in our study.

LncRNAs, an important epigenetic factor, could function in the reproductive process through cis and trans regulating gene activity. In the current study, a total of 3164 lncRNAs were identified. Fewer lncRNAs were identified than that in chicken brain (40), adipose tissue and liver (41), indicating tissue specific expression patterns and characteristics of lncRNAs. Additionally, the identified lncRNAs exhibited fewer exons, shorter transcripts, which is consistent with studies in other species (42). Consistent with previous research, intergenic lncRNAs was the most abundant class of lncRNAs in the genome (43, 44).

The number of DEGs and DELs between two parental lines was greater than that between crossbreeds and their purebreds, indicating the genetic difference between two purebreds was larger than any other groups. Similar results were also revealed by the previous study (45). Genome heterozygosity arising from genomics sequence variation between two purebreds is one fundamental driving force for the phenotype heterosis. As in the case of dominant or recessive allele pairs in classical genetics, the dominance expression of genes resulted in a nonlinear phenotypic effect of alleles at one locus (46). Therefore, nonadditive expression pattern is important for the formation of heterosis. Wu et al. (47) reported that dominant genes involved in carbohydrate metabolism were associated with heterosis for body weight in Drosophila melanogaster. It was also found the nonadditive genes may drive the formation of heat stress heterosis in cattle (48). In chicken, the nonadditive genes were illustrated to involved in oxidative phosphorylation, contributing to breast muscle mass heterosis (12). In the present study, most DEGs and DELs possessed nonadditivity pattern in hybrids, indicating nonadditivity was the critical gene expression pattern affecting traits heterosis. Functional enrichment analysis showed that nonadditive genes were associated with animal organ development, female gonad development, ECM-receptor interaction and GnRH signaling pathway, such as *laminin subunit alpha 4* (*VTN*), *CD36 molecule* (*CD36*) and *fibronectin 1* (*FN1*), which were revealed to function in ovary cell growth and proliferation (49–51) and specifically expressed in crossbreeds’ ovary. In addition, the nonadditive gene *purinergic receptor P2X 1* (*P2RX1*) was identified, which was enriched in calcium signaling pathway. *P2RX1* was demonstrated as potential candidate genes for egg production of ducks (52). Hence, we hypothesized these biological process and pathways might involve in the formation of sexual maturation heterosis through modulating gene expression.

The underlying mechanism of sexual maturation heterosis is complicated. WGCNA may be an effective method for mining valuable expression data to analyze the intricate genetic networks (35). By focusing on the association between co-expression modules and traits of interest, we identified seven modules were significantly correlated with pubic space and oviduct length. Genes harbored in 7 modules were enriched in GnRH signaling pathway, ECM-receptor interaction, calcium ion binding and tissue development, which were similar to the enrichment results of nonadditive genes. The overlapped biological processes may play more crucial role in the formation of sexual maturation heterosis.

The gonad development is part of prenatal development of the reproductive system and ultimately forms the ovary in the female (53). In this study, female gonad development was significantly enriched by nonadditive genes including *CEBPB*, *COL6A1*, *TGFB1* and *KIT*. Previous studies demonstrated that *CEBPB, COL6A1* and *KIT* may be essential for female reproduction through regulating the cell development, ovulation and luteinization of ovarian follicle (54–56). *TGFB1* is implicated as a key regulator of the development and cyclic re-modelling characteristic of reproductive tissues (57). Altered plasma *TGFB1* has been found closely associated with reproductive process in female (58). The up-regulated TGFB1 contributed follicles development and ovulation in sheep (44). In our study, the expression of *TGF*B*1* was the lowest in YY, and significantly associated with oviduct length, suggesting *TGFB1* may act an indicator to estimate the heterosis of sexual maturation. Additionally, previous studies found several ovarian lncRNAs could involve in follicular development by regulating target genes related to *TGF-β* in small-tailed sheep (59, 60). In the current study, *TGFB1* were found significantly correlated with oviduct length, and regulated by *MSTRG.17017.1* and *MSTRG.6475.20* in trans. *MSTRG.17017.1* and *MSTRG.6475.20* were both novel lncRNAs in chicken. These indicated the two pairs of mRNAs and lncRNAs could be critical candidates for regulating the formation of sexual maturation heterosis. The putative function of candidate genes performed in the development and sexual maturation, supporting direct cue for nonadditive genes involved in sexual maturation heterosis.

GnRH signaling is regarded as the gonadotrope and endocrine control of fertility in mice (61) and goose (62). In chicken, GnRH signaling pathway could involve in ovarian function (49, 63). Here, the nonadditive genes, such as *MMP2*, *ADCY6*, *EGR1*, *CACNA1C* and *GNAQ*, were identified significantly enriched in GnRH signaling pathway. Importantly, *GNAQ* regulates estrus in sheep by controlling GnRH secretion and release through calcium signaling pathway (64). In ovary, *GNAQ* can also increase the size of ovarian follicles (65). Similarly, *CACNA1C*, associated with calcium channel function and crucial for onset of the reproductive maturation process (66). These two genes were involved in GnRH signaling pathway, implying they may act upstream factors to affect the downstream genes involved in gonad development. These results supported GnRH signaling pathway could involve the sexual maturation heterosis. Moreover, several lncRNAs were also evidenced involving in spermatogenesis and the time of puberty through regulating their target genes in bull (67). Herein, the expression differences of *GNAQ* and *CACNA1C* were regulated by *MSTRG.19756.2* in trans. *MSTRG.19756.2* was novel lncRNA. Our findings imply ovary *MSTRG.19756.2* may be associated with reproductive impairment by controlling their target genes (*GNAQ*, *CACNA1C*) related with the GnRH signaling pathway. Admittedly, sexual maturation is initiated by the release of GnRH (68). The GnRH and MT content were further detected, and confirmed the results of gene expression. Upon light stimulation, decreasing levels of MT results in a decrease in GnIH synthesis (69), thus alleviating the inhibition on the HPG axis and allowing for the release of GnRH and subsequent activation of gonad development (70). Consistent with the previous study (71), we found that chickens with earlier AFE and larger pubic space had higher GnRH content but lower MT content, suggesting that chickens with higher GnRH could give impetus to development of reproductive tissue, contributing to the sexual maturation in crossbreeds. Interestingly, the crossbreeds shared same photoperiod stimulus with purebreds, but exhibited significant heterosis for sexual maturation. Hence, we speculated the disparity of light perception and the differences between crossbreeds and purebreds may drive the formation of sexual maturation heterosis (72). This may provide novel insights into understanding the neuroendocrine neurons controlling the onset of puberty and fertility in mammals including humans.

## Conclusions

Our study focused on revealing the phenomenon of heterosis in chickens, and positive heterosis of sexual maturation was observed. Genome-wide gene expression pattern analysis showed that nonadditivity was the critical mode of mRNAs and lncRNAs action for sexual maturation heterosis. The nonadditive lncRNAs *MSTRG.6475.20* and *MSTRG.17017.1* and their target nonadditive genes (*GNAQ*, *CACNA1C* and *TGFB1*), which involved in GnRH signaling pathway and female gonad development, played a critical role in driving the formation of sexual maturation heterosis. Our findings would provide novel insight into the molecular basis of positive heterosis for sexual maturation in chicken and theoretical basis for improving egg production.

## Data Availability Statement

The transcriptome data are available in the Sequence Read Archive (https://www.ncbi.nlm.nih.gov/sra) at NCBI, with the BioProject ID: PRJNA845127 and SRA Accession Number: SAMN28857461-28857484.

## Ethics Statement

This study was carried out in line with the Guidelines for Experimental Animals established by the IAS-CAAS (IAS2022-35).

## Author Contributions

JC, YS and JY conceived and designed the project. JY and YW performed bioinformatics analyses, YW conducted the experiments and drafted the manuscript. YW, AN, YZ, JZ, SB and YL participated in animal manipulation and data collection. PW, LS, YL and HM discussed the manuscript. All authors contributed to sample collecting and approved the submitted version. All authors contributed to the article and approved the submitted version.

## Funding

This research was funded by the National Natural Science Foundation of China (32172721), China Agriculture Research System (CARS-40), the Fundamental Research Funds for Central Non-profit Scientific Institution (2021-YWF-ZYSQ-12), and the Agricultural Science and Technology Innovation Program (ASTIP-IAS04).

## Conflict of Interest

The authors declare that the research was conducted in the absence of any commercial or financial relationships that could be construed as a potential conflict of interest.

## Publisher’s Note

All claims expressed in this article are solely those of the authors and do not necessarily represent those of their affiliated organizations, or those of the publisher, the editors and the reviewers. Any product that may be evaluated in this article, or claim that may be made by its manufacturer, is not guaranteed or endorsed by the publisher.

## Supplementary Material

Supplementary Figure 1 Chromosomal distribution of putative and known lncRNAs.

Supplementary Figure 2 Principal components analysis (PCA) of identified and genes in the crossbreeds (WY, YW) and purebreds (WW, YY).

Supplementary Figure 3 The Venn analysis of nonadditive lncRNAs and genes in WY and YW. (A) The Venn analysis of nonadditive lncRNAs in WY and YW. (B) The Venn analysis of nonadditive genes in WY and YW.

Supplementary Figure 4 The expression of FSHR and ESR2 in different groups.

Supplementary Table 1 Summary statistics for transcriptome sequencing data, the identified lncRNAs.

Supplementary Table 2 The results of differentially expressed lncRNAs and differentially expressed genes in different groups.

Supplementary Table 3 Classification of different expression patterns of mRNAs and lncRNAs.

Supplementary Table 4 The enriched GO terms and pathways of nonadditive genes and detailed information of the shared nonadditive genes enriched in key biological process.
