# Supplementary figures and images for: Genetic Basis of Sexual Maturation Heterosis: Insights From Ovary lncRNA and mRNA Repertoire in Chicken
Source: Front Endocrinol (Lausanne). 2022 Jul 27;13:951534. doi: 10.3389/fendo.2022.951534 (PMC9363637; doi:10.3389/fendo.2022.951534)

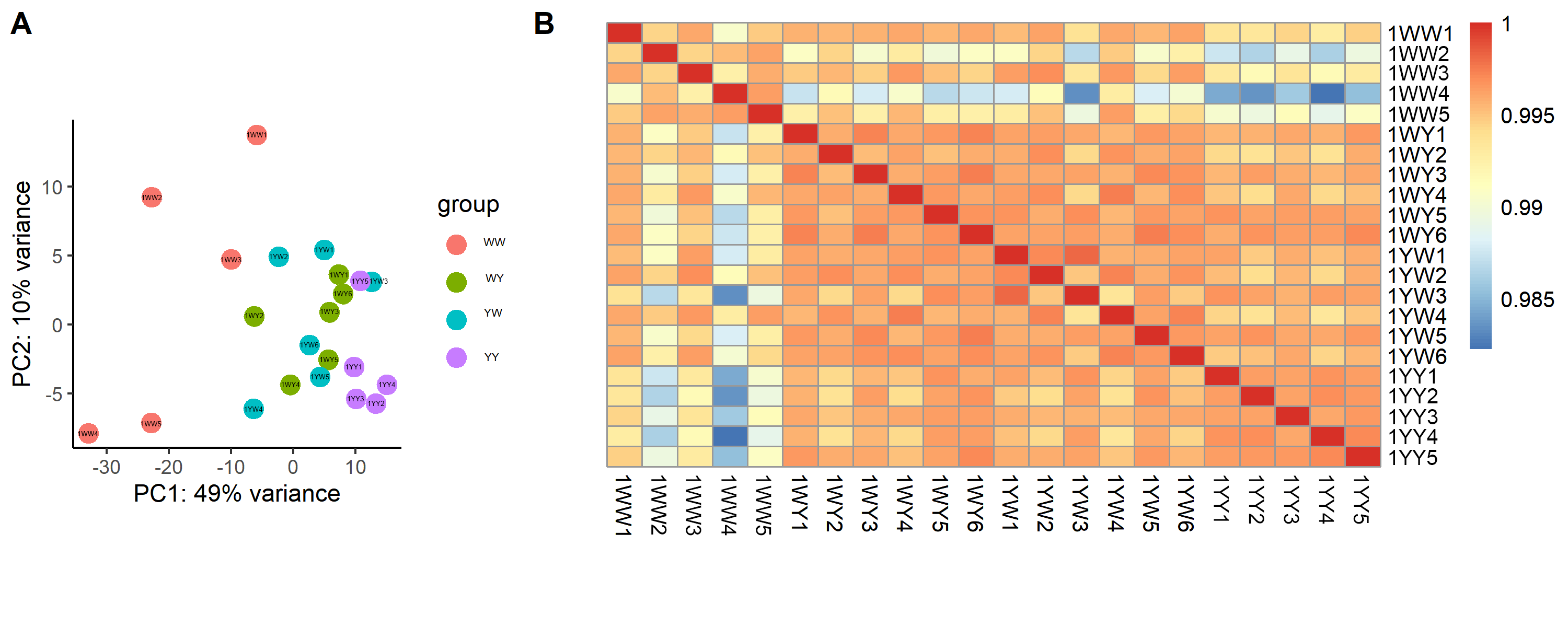

Supplement: Supplementary Figure 2 — Principal components analysis (PCA) of identified and genes in the crossbreeds (WY, YW) and purebreds (WW, YY). [file Image_2.tif]

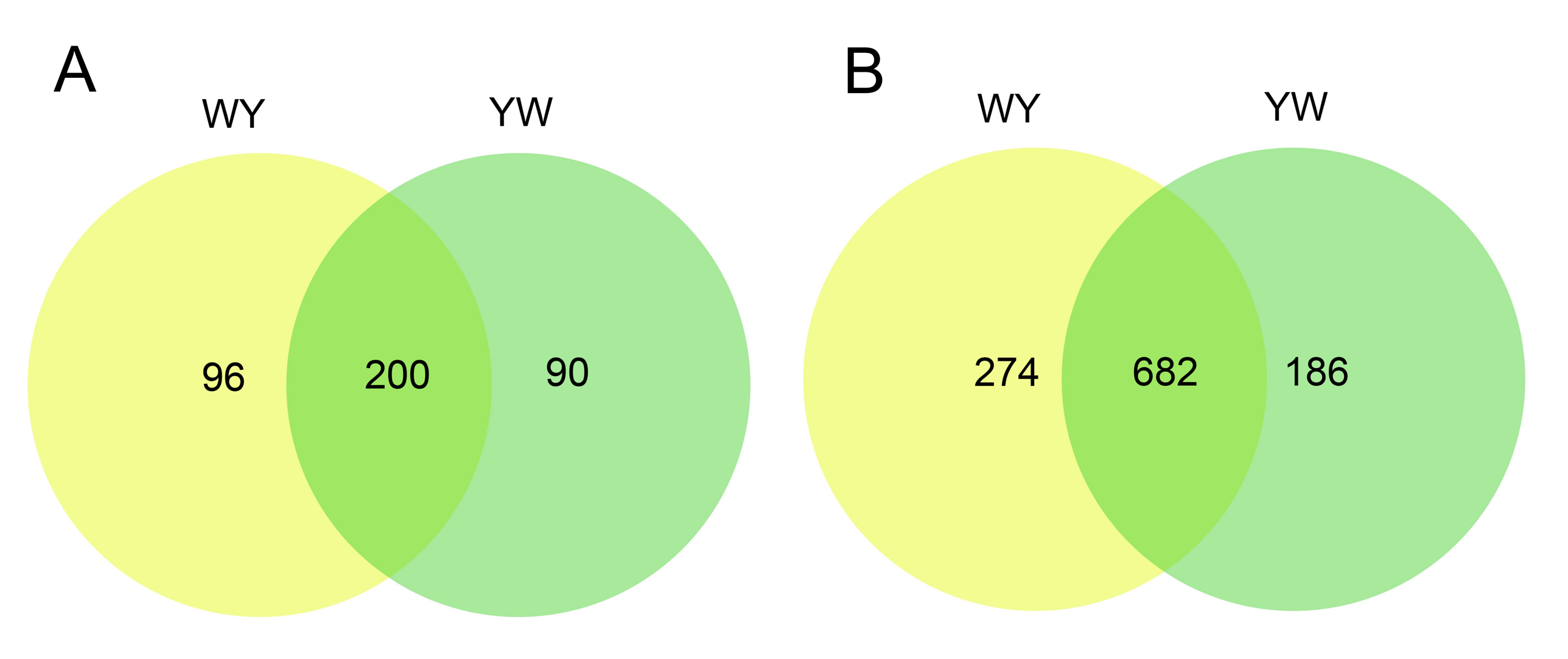

Supplement: Supplementary Figure 3 — The Venn analysis of nonadditive lncRNAs and genes in WY and YW. (A) The Venn analysis of nonadditive lncRNAs in WY and YW. (B) The Venn analysis of nonadditive genes in WY and YW. [file Image_3.tif]
